# Flagellar apparatus structure of choanoflagellates

**DOI:** 10.1186/s13630-016-0033-5

**Published:** 2016-05-04

**Authors:** Sergey A. Karpov

**Affiliations:** Zoological Institute RAS, Universitetskaya emb. 1., St. Petersburg, 199034 Russia; St. Petersburg State University, Universitetskaya emb. 7/9., St. Petersburg, 199034 Russia

**Keywords:** Choanoflagellates, Flagellar apparatus, Basal body, MTOC, Central filament

## Abstract

Phylum choanoflagellata is the nearest unicellular neighbor of metazoa at the phylogenetic tree. They are single celled or form the colonies, can be presented by naked cells or live in theca or lorica, but in all cases they have a flagellum surrounded by microvilli of the collar. They have rather uniform and peculiar flagellar apparatus structure with flagellar basal body (FB) producing a flagellum, and non-flagellar basal body (NFB) lying orthogonal to the FB. Long flagellar transition zone contains a unique structure among eukaryotes, the central filament, which connects central microtubules to the transversal plate. Both basal bodies are composed of triplets and interconnected with fibrillar bridge. They also contain the internal arc-shaped connectives between the triplets. The FB has prominent transitional fibers similar to those of chytrid zoospores and choanocytes of sponges, and a radial microtubular root system. The ring-shaped microtubule organizing center (MTOC) produces radial root microtubules, but in some species a MTOC is represented by separate foci. The NFB has a narrow fibrillar root directed towards the Golgi apparatus in association with membrane-bounded sac. Prior to cell division, the basal bodies replicate and migrate to poles of elongated nucleus. The basal bodies serve as MTOCs for the spindle microtubules during nuclear division by semiopen orthomitosis.

## Body of the primer

Choanoflagellates: *Monosiga ovata*, *Codosiga botrytis, Desmarella thienemanni, Salpingoeca* sp.

**Fig. 1:**
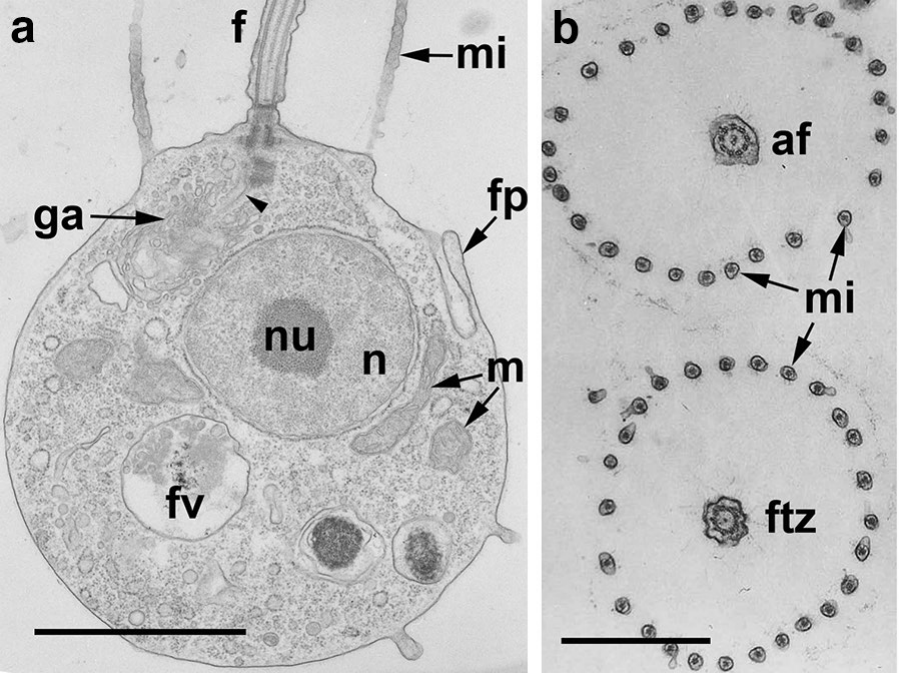
General organization of *M. ovata.* **a** Longitudinal section of the cell. **b** Transversal sections of the collar of two neighbor cells at the axoneme level (*top*), and the transition zone (*down*). *af* axoneme of flagellum, *f* flagellum, *fp* food pseudopodium, *ftz* transition zone of flagellum, *fv* food vacuole, *ga* golgi apparatus, *m* mitochondrium, *mi* microvilli, *n* nucleus, *nu* nucleolus. *Scale bars* **a** 1.5 µm, **b** 1 µm

Choanoflagellates form a monophyletic group sister to Metazoa (what was predicted by James-Clark nearly 150 years ago [5]), and split into two branches: marine loricate Acanthoecida and naked or thecate Craspedida [2, 22]. The choanoflagellate has one flagellum surrounded by microvilli of the collar, central nucleus, dictyosome of Golgi apparatus, which is in between the nucleus and basal bodies and connected to them with thin fibrillar root (Fig. 1). Mitochondria with flat cristae are located around the nucleus; food vacuoles are in the basal part of the cell.

The representatives of Craspedida, *M. ovata*, *C. botrytis*, *D. thienemanni* and *Salpingoeca* sp., are better studied in respect of flagellar apparatus structure. They have rather uniform flagellar apparatus, composed of one flagellum and two basal bodies, as well as all the other choanoflagellates, differing from each other by the microtubular root organization.

**Fig. 2:**
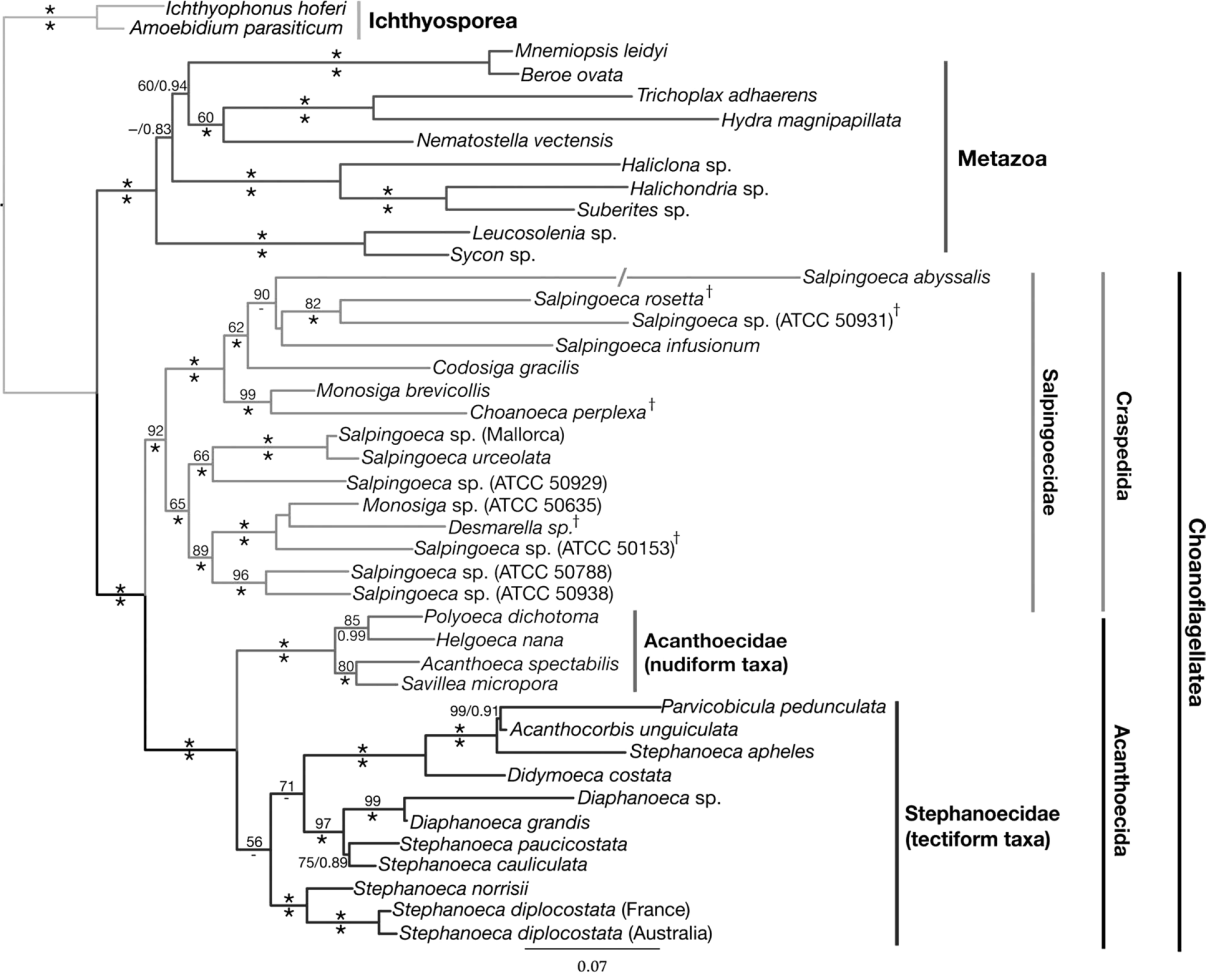
Molecular phylogenetic tree of choanoflagellates based on a concatenated dataset of small and large subunit rDNA, hsp90, and tubA genes. The choanoflagellata form a sister group to metazoa and divided into two main clusters: the Craspedida including the naked and thecate, marine and freshwater forms, and the Acanthoecida—marine loricate forms. (After Ref. [22])

Precise position of each craspedid sequence in the phylogenetic tree is not well supported (Fig. 2); therefore, we do not discuss here the phylogeny of mentioned above genera and species among the Craspedida.

## Basal body structure

The flagellar apparatus of choanoflagellates is composed of one flagellum and two orthogonal basal bodies (flagellar and non-flagellar ones) producing the microtubular and fibrillar roots. Both basal bodies are mainly similar to each other, contain triplets of microtubules.

**Fig. 3:**
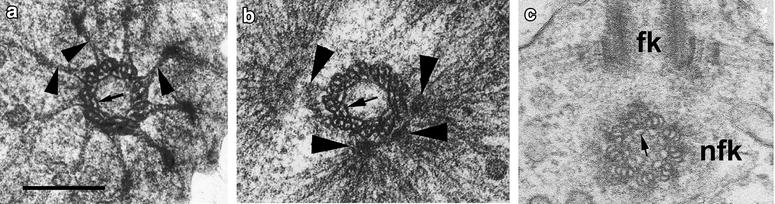
Structure of flagellar basal body in *D. thienemanni* (**a**, **b**) and non-flagellar basal body in *M. ovata* (**c**). **a** Transversal section of the FB distal end. *Arrowheads* show the prominent transitional fibers, connecting basal body to the plasma membrane. Note the *arc-shaped* filaments (*arrow*) inside basal body, interconnecting neighbor triplets. **b** Transversal section of FB middle part. *Arrow* shows the arc-shaped filaments inside FB; *arrowheads* point 4 separate MTOCs initiating the microtubular roots. **c** Transversal section of NFB (nfk). *fk* flagellar basal body. **a**, **b** after: [8]; **c** after: [11]. *Scale bar* 200 nm

The distal end of the *flagellar basal body* (FB) is connected to the cell plasma membrane with nine well-developed transitional fibers, which seem to be connected with internal arc-shaped connectives of basal body (Fig. 3a, b). Each transitional fiber (appr. 160 nm in length) is composed of two threads starting from A to C tubules correspondingly and fusing at the dense granule on the plasma membrane (Fig. 3a). The internal arc-shaped connectives interconnect the neighbor triplets in the middle and distal part of FB of *D. thienemanni* (Fig. 3a, b).

**Fig. 4:**
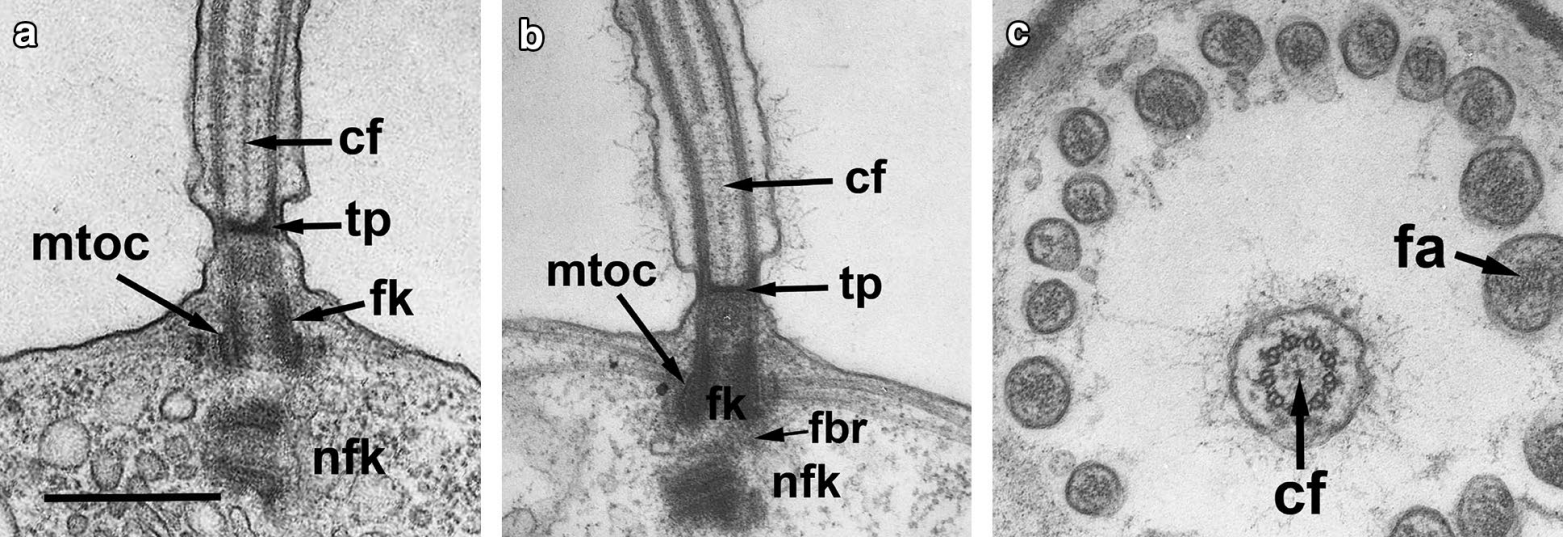
Structure of flagellar transition zone and root system in *M. ovata* (**a**) and *Salpingoeca* sp. (**b**, **c**). **a**, **b** Longitudinal section of flagellar apparatus with FB (fk) and NFB (nfk) having orthogonal orientation. **c** Transversal section of flagellar transition zone and microvilli of the collar. *cf* central filament, *fa* bandle of f-actin inside microvillus, *fbr* fibrillar bridge interconnecting basal bodies, *fk* flagellar basal body, *mtoc* electron dense ring around the FB (fk), producing radial root microtubules, *nfk* non-flagellar basal body, *tp* transversal plate. **a** after: [11]. *Scale bar* 500 nm

**Fig. 5:**
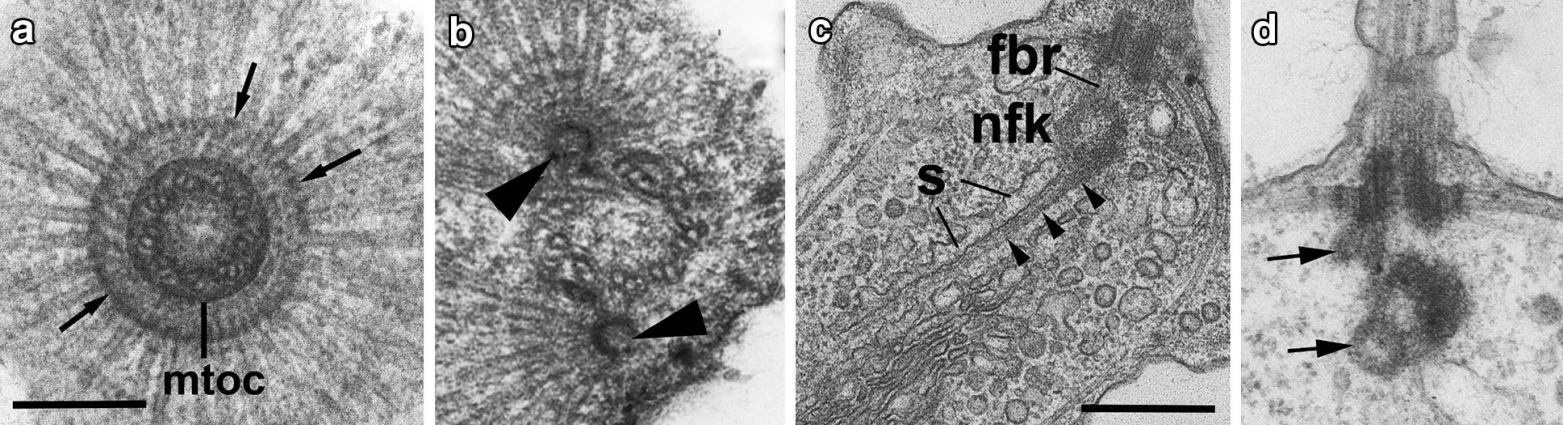
Structure of microtubular roots (**a**, **c**) and basal bodies replication (**d**) in *M. ovata,* and microtubular roots in *Sphaeroeca volvox* (**b**). **a**, **b** Transversal section of FB at its proximal end with MTOCs and radiating microtubules. *Arrows* show the electron dense interstitial material, *arrowheads*—two separate MTOCs. **c** Longitudinal section of flagellar apparatus showing the fibrillar root (*arrowheads*) associated with membrane sac (s). **d** Longitudinal section of flagellar apparatus prior cell division, showing nascent basal bodies (*arrows*). *fbr* fibrillar bridge interconnecting basal bodies, *nfk* non-flagellar basal body, *nu* nucleolus, **a**, **c** after: [11]; **b** after: [6]. *Scale bars* **a**, **b** 200 nm; **C**, **D** 500 nm

The *non-flagellar basal body* (NFB) also contains similar internal connectives, but does not have the transitional fibers (Fig. 3c). The NFB is located at approximately right angles to the flagellar base and is connected to it by a fibrillar bridge (Figs. 4b, 5c). The latter connects one edge of FB proximal end to the upper surface of the broader end of the NFB.

There is *no centrosome* in the choanoflagellate cell. Thus, the basal bodies serve as microtubular organizing centers during the nuclear division, which belongs to semiopen orthomitosis [10, 12].

*The long transition zone* contains a transversal plate located above the cell surface. The central two microtubules within the flagellar transition zone are replaced by a single central filament of some considerable length, which connects the transversal plate with central tubules (Fig. 4). Sometimes the thickness in the center of transversal plate is present. A central filament has been described for the first time by D. Hibberd in *C. botrytis* [4] and then was found in all investigated choanoflagellates [6–8, 13, 18]. This central filament is unique for choanoflagellates as no such structure was found in the flagellar transition zone of other eukaryotes [9].

The space between FB and transversal plate is filled with amorphous material (Figs. 1, 4), which was found in all investigated flagellar apparata of choanoflagellates [18, 25].

### Flagellar roots

In *M. ovata,* the proximal portion of flagellar basal body is surrounded by a ring of electron dense material from which the root, containing approximately 60 radially arranged microtubules, originates (Fig. 5a). In the immediate region of the flagellar base, the microtubules are stacked in two layers and two additional rings of electron dense material fill the spaces between the microtubules (Fig. 5a). From the flagellar base, the microtubules pass outwards and laterally just beneath the plasma membrane for about one-third or half the length of the cell (Figs. 1, 5c). They are probably responsible for shaping the apical end of the cell. The organization of the root microtubules around the FB is the same in many members of the Acanthoecida and *Salpingoeca* [11]. But the root organization in the naked Craspedida is more complex with the radial microtubules converging on 4–5 foci in *C. botrytis* [4] and *D. thienemanni* (Fig. 3b) and on two cylinders in *Sphaeroeca volvox* (Fig. 5b).

From the lower surface of NFB, a long, narrow, slightly striated, fibrillar root passes obliquely towards the Golgi apparatus (Figs. 1, 5c). It is associated with one surface of a membrane-bounded sac which extends from the region of the flagellar base and passes to about half way along the dictyosome. This fibrillar-membrane complex is closely associated with the dictyosome and its orientation appears to be determined by the relative position of the dictyosome with respect to the nucleus. In *M. ovata*, the fibrillar root can either be directed away from the end of the NFB or deflected backwards underneath the flagellar base.

The fibrillar bridge between the two basal bodies and the narrow striated fibrillar root passing in Golgi apparatus direction and closely applied to a membrane sac is probably present in all choanoflagellates [11].

## Basal body life cycle and other functions

New basal bodies appear on the base of the old ones before cell division. Each basal body produces the nascent basal body (Fig. 5d). Then the flagellum is retracted into the cell and the axoneme is disassembled. The pairs of basal bodies each composed from the old and the new one migrate from each other to the poles of the prophases’ nucleus [10, 12]. Which basal body (the old or the new one) produces a new flagellum in the daughter cell is not clear at the moment. Both basal bodies present in the cyst of *M. ovata* [19]. Thus, the FB and NFB present at all studied stages of the choanoflagellate life cycle function as a centrosome during mitosis.

Genomic studies of *Monosiga brevicollis* [14] and *Salpingoeca rosetta* [3] did not discuss basal body components.

## Notable basal body findings

The notable finding about basal bodies of choanoflagellates is the central filament in the flagellar transition zone—a unique structure among eukaryotes. Another feature, which is also rare in protists, is a radial microtubular system. The differences in microtubular root organization reflect the peculiarities of choanoflagellates at the genus level [6, 11]. The prominent transition filaments are the characters of flagellar apparatus of chytridiomycete zoospores [1], and of the sponge choanocytes [23, 24], but the internal arc-shaped connectives in both the FB and NFBs have been found in the choanoflagellates only [8].

## Future of basal body research in choanoflagellates

The flagellar apparatus structure has been studied in details in craspedid freshwater choanoflagellates [11, 13, 21]. Such information on marine representatives is rather poor at the moment [15–17, 20, 21] because of difficulties with fixation of marine cells. The central filament in the transition zone has been found in marine *Stephanoeca diplocostata* [18], but the other peculiarities are not clear. According to our general dogma, that the marine choanoflagellates are more ancient than the freshwater ones, we can propose that close attention to the flagellar apparatus of marine choanoflagellates can give new unexpected knowledge on the flagellar apparatus characters.
